# Genomic alterations predictive of poor clinical outcomes in pan-cancer

**DOI:** 10.18632/oncotarget.28276

**Published:** 2022-09-28

**Authors:** Crystal S. Seldon, Karthik Meiyappan, Hannah Hoffman, Jimmy A. Guo, Neha Goel, William L. Hwang, Paul L. Nguyen, Brandon A. Mahal, Mohammed Alshalalfa

**Affiliations:** ^1^Sylvester Comprehensive Cancer Center, University of Miami, Miami, FL 33136, USA; ^2^Miller School of Medicine, University of Miami, Miami, FL 33136, USA; ^3^Department of Radiation Oncology, Massachusetts General Hospital, Boston, MA 02114, USA; ^4^Broad Institute of MIT, Cambridge, MA 02142, USA; ^5^School of Medicine, University of California San Francisco, San Francisco, CA 94143, USA; ^6^Dana-Farber Cancer Institute and Brigham and Women’s Hospital, Boston, MA 02215, USA; ^*^Co-first authors

**Keywords:** genomic alterations, TP53, TCGA, poor prognosis

## Abstract

Background: Genomic alterations are highly frequent across cancers, but their prognostic impact is not well characterized in pan-cancer cohorts. Here, we use pan-cancer cohorts from TCGA and MSK-IMPACT to evaluate the associations of common genomic alterations with poor clinical outcome.

Materials and Methods: Genomic alterations in commonly altered genes were extracted from Pan-Cancer TCGA and MSK-IMPACT cohorts. Multivariable Cox regression analyses stratified by cancer type defined adjusted hazard ratios (AHRs) for disease-specific survival (DSS), progression-free survival (PFS) and overall survival (OS).

Results: Using TCGA we identified 32 mutated genes, and 15 copy number (CN) genes with frequency >= 4% in 9,104 patients across 28 cancers. On UVA, having a TP53-mutations or any mutation in the 31 genes (mut31) were associated with worse PFS (HR: 1.22, *p* < 0.0001 and HR: 1.1, *p* = 0.04, respectively) and DSS (HR: 1.38, *p* < 0.0001, and HR: 1.16, *p* = 0.03, respectively). CDKN2A, PTEN deletions, and MYC-amplifications were associated with PFS and DSS (*p* < 0.05 for all).

On MVA, including TP53-mutations, mut31, CDKN2A-deletion, PTEN-deletion, and MYC-amplification, all five alterations were independently prognostic of poor PFS and DSS.

Similar results were observed in an independent cohort from MSK-IMPACT (*n* = 7,051) where TP53 was associated with poor OS independent of mut31 and CN alterations in CDKN2A, PTEN, and MYC in primary tumors (*p* < 0.0001).

Conclusions: TP53-mutations, CDKN2A-deletion, PTEN-deletion, and MYC-amplification are independent pan-cancer prognostic genomic alterations.

## INTRODUCTION

There is growing interest in genomic profiling (i.e. tumor mutations, copy number (CN) alterations) for cancer therapy and precision oncology to inform treatment decisions and identify patients for relevant clinical trials [1].

Numerous cancer institutes have adopted genomic profiling into clinical practice, generating large-scale datasets that lend themselves to subsequent exploratory analyses [2]. Earlier studies from MSK-IMPACT study represents a first step towards evaluating the clinical impact of large-scale prospective tumor sequencing [3]. TP53 was the most frequently mutated gene in MSK-IMPACT (41.7% of cases), followed by KRAS (15%), TERT (13%), and PIK3C (12%). CDKN2A was the most frequently deleted gene (7%), while CCND1 (4.3%), MYC (4%), and ERBB2 (4%) were the most frequently amplified genes.

Several studies have characterized the association of TP53 mutations with clinical outcomes in breast cancer [4], non-small-cell lung cancer [5], cholangiocarcinoma [6], thymic carcinoma [7], and in pan-cancers [8, 9]. Other studies have demonstrated that gaining additional mutations or CN alterations increases risk of disease progression. For example, men with prostate tumors that have compound tumor suppressor gene mutations (TP53, PTEN, RB1) have poorer outcomes [10]. Although previous research has elucidated the prognostic significance of TP53 mutations in individual cancers, the independent prognostic role of mutations in this gene at a pan-cancer level independent of other mutations and genomic alterations remains unclear.

In this work, we identified the most commonly mutated and copy number altered genes from the Pan-Cancer TCGA cohort (*n* = 9,104) and associated them with progression free survival (PFS) and disease specific survival (DSS) independently and after adjusting for TP53 mutations. We subsequently used the MSK-IMPACT cohort (*n* = 7,051) to validate the associations between genomic alterations and overall survival (OS) in patients with primary and metastatic tumors.

## RESULTS

### Baseline characteristics

The TCGA cohort included 9,104 patients across 28 cancer types, with clinical outcome data in 7,465 of these patients. The MSK-IMPACT (validation cohort) included 7,051 patients across 12 cancer types, 3,684 patients had non-metastatic cancer and 3,367 had metastatic cancer.

### Common pan-cancer genomic alterations in the Pan-Cancer TCGA pan-cancer cohort

TCGA identified 32 mutated genes (including TP53) and 15 CN altered genes with frequency greater than or equal to 4% (≥4%) in 9,104 patients across 28 cancers. In the TCGA cohort, 37% of subjects had a TP53 mutation, and 68% had at least one mut31 mutation; furthermore, 81% of patients with TP53 mutations had one additional mutation in at least one of the mut31 genes. Furthermore, CDKN2A was the most frequent deletion (10.9%) and MYC was the most frequent amplification (13%) (Supplementary Table 1).

### Prognostic impact of common genomic alterations in cancer (Pan-Cancer TCGA cohort)

**Figure 1:**
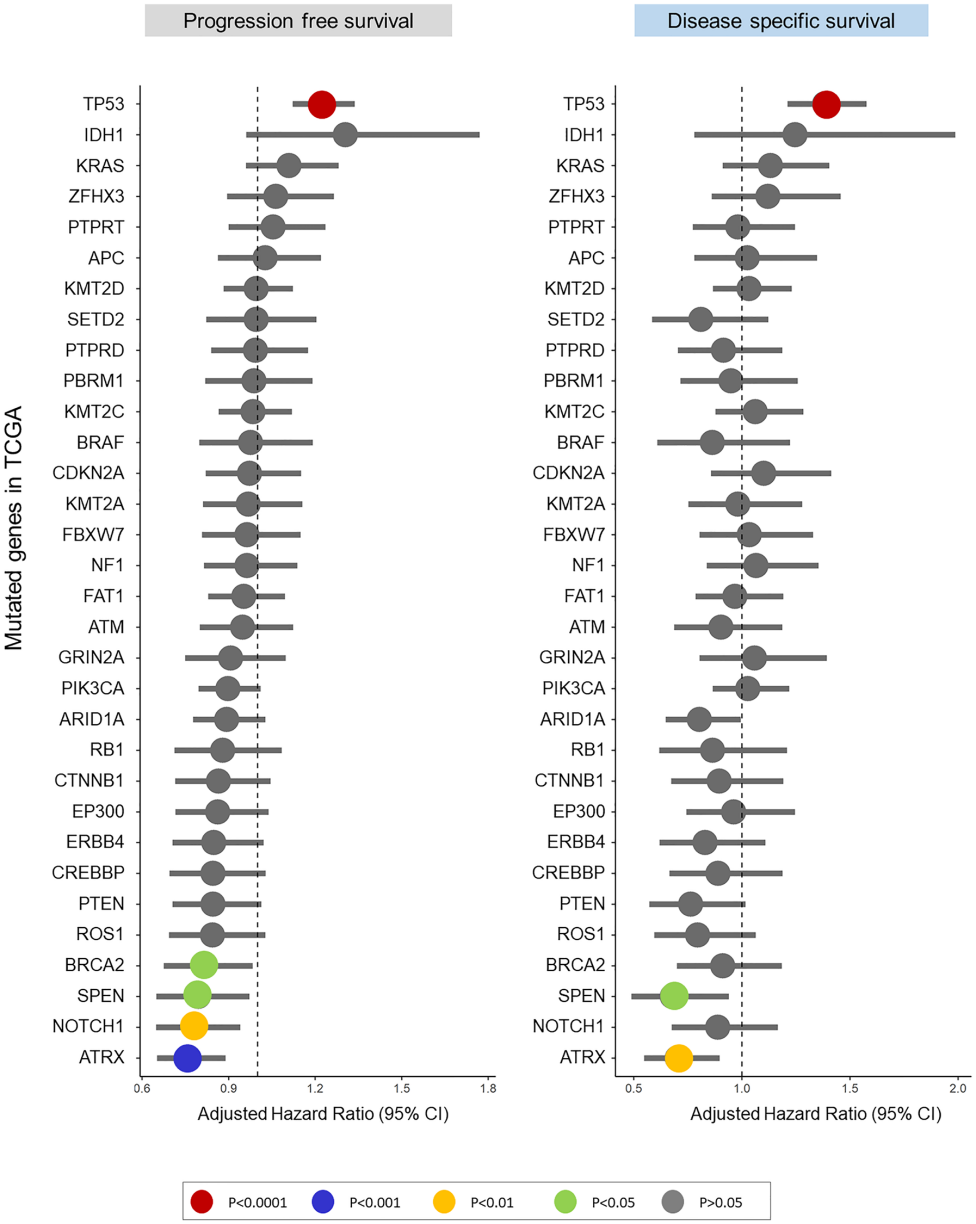
Adjusted hazard ratios from stratified Cox regression model predict PFS and DSS in the Pan-Cancer TCGA cohort for the 32 most common mutations.

**Figure 2:**
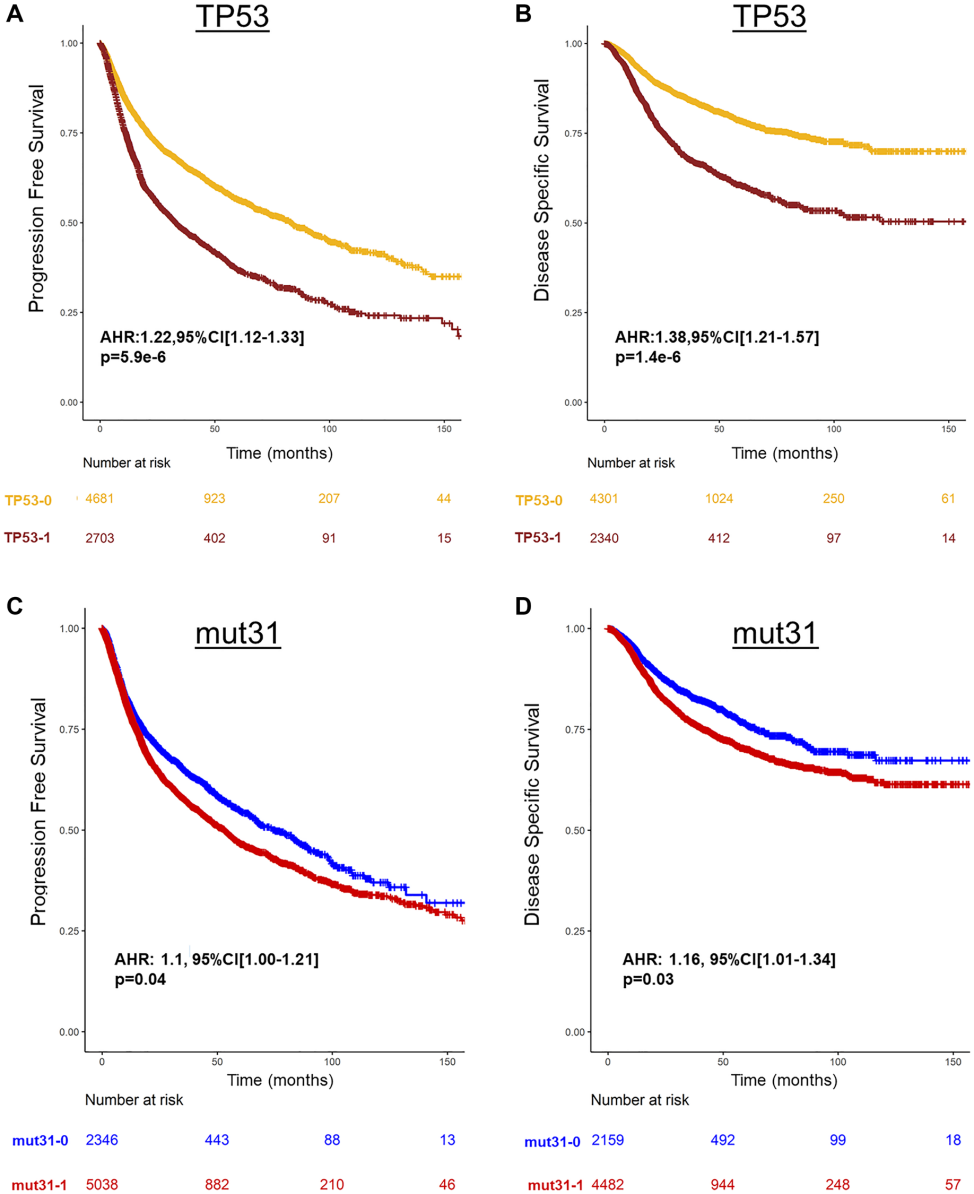
Prognosis of TP53 and mut31 in Pan-Cancer TCGA. (**A**, **B**) Survival curves of TP53 prognostic of poor PFS and DSS, (**C**, **D**) and mut31 is prognostic of PFS and DSS. Adjusted Hazard Ratios (AHR) are derived from Cox regression stratified by cancer type.

Across all 32 genes, only TP53 mutations were associated with poor PFS and DSS (Figure 1, Supplementary Table 2). On Univariable analysis (UVA), TP53 mutations alone or mutations in any one of the other 31 genes (mut31) were associated with worse PFS (HR: 1.22, 95% CI [1.12–1.33], *p* = 5.9e^−6^ and HR: 1.1, 95% CI [1.00–1.21], *p* = 0.04, respectively) and DSS (HR: 1.38, 95% CI [1.21–1.57], *p* = 1.4e^−6^ and HR: 1.16, [1.01–1.34], *p* = 0.03, respectively) (Figure 2A–2D, Supplementary Table 2). Notably, patients with TP53 missense mutations had similar PFS and DDS to patients with truncating mutations (*p* = 0.3, *p* = 0.56 respectively). On Multivariable analysis (MVA), including TP53 and mut31 in the model, only TP53 was prognostic of PFS (HR: 1.21, 95% CI [1.11–1.33], *p* = 1.3e^−5^) and DSS (HR: 1.37, 95% CI [1.2–1.56], *p* = 3.6e^−6^), whereas mut31 was not prognostic. Moreover, none of the 31 genes was prognostic of PFS or DSS when adjusting for TP53 mutations (Supplementary Table 3). There were 4 genes which were prognostically protective for DSS, BRCA2, SPEN, TORCH1, and ARTX. When analyzing the same mut31 signature but without these 4 genes in a separate signature (mut27), there was no difference in its ability to predict prognosis. Additionally, both mut31 and mut27 were not prognostic when adjusted for TP53.

Among patients with TP53 mutations, mut31 was not associated with PFS (HR: 0.98, 95% CI [0.84–1.15], *p* = 0.82) or DSS (HR: 1.17, 95% CI [0.93–1.47], *p* = 0.16). When mut31 was stratified into five groups (no mutations in mut31: mut31-0, one mutation: mut31-1, two mutations: mut31-2, three to five mutations: mut31-(3-5), more than five mutations: mut31->5), only mut31-(3-5) patients with a TP53 mutation had an increased risk of DSS (Supplementary Table 4). Thus, additional mutational burden within the mut31 genes for patients already containing a TP53 mutation were not associated with a ‘dose-response’ relationship for DSS or PFS.

Fraction of genome altered was also investigated. When including this in the MVA of TP53 and mut31, TP53 remained independently prognostic of PFS (HR: 1.15 [1.04–1.27], *p* = 0.0038) and DSS (HR: 1.33 [1.15–1.53], *p* = 8e-5). The fraction of genomic alteration was also prognostic (*p* < 0.001) for both endpoints, but mut31 was not prognostic.

**Figure 3:**
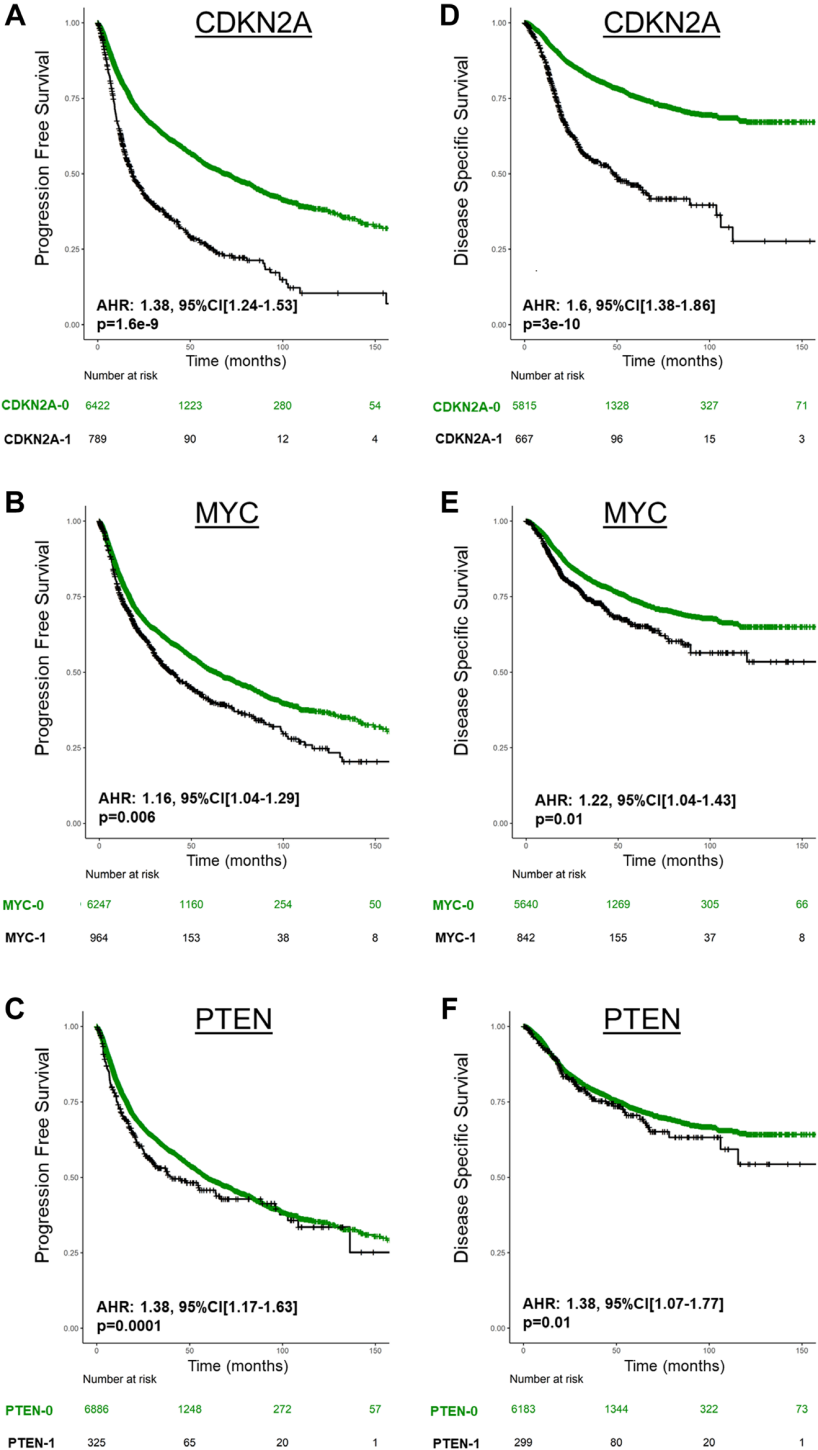
Prognosis of CDKN2A deletion (CDKN2A-1), MYC amplification (MYC-1), and PTEN deletion (PTEN-1) in Pan-Cancer TCGA for PFS (**A**, **B**, **C**) and DSS (**D**, **E**, **F**). AHR are derived from Cox regression stratified by cancer type.

When characterizing the prognostic impact of CN on UVA, only CDKN2A deletion, PTEN deletion, and MYC amplification were significantly associated with poor PFS and DSS (*P* < 0.05 for all, Figure 3, Supplementary Figure 1). On MVA adjusting for TP53 mutations, CDKN2A deletion, PTEN deletion, and MYC amplification were the only CN altered genes associated with poor PFS and DSS (*P* < 0.05 for all, Supplementary Table 5).

**Figure 4:**
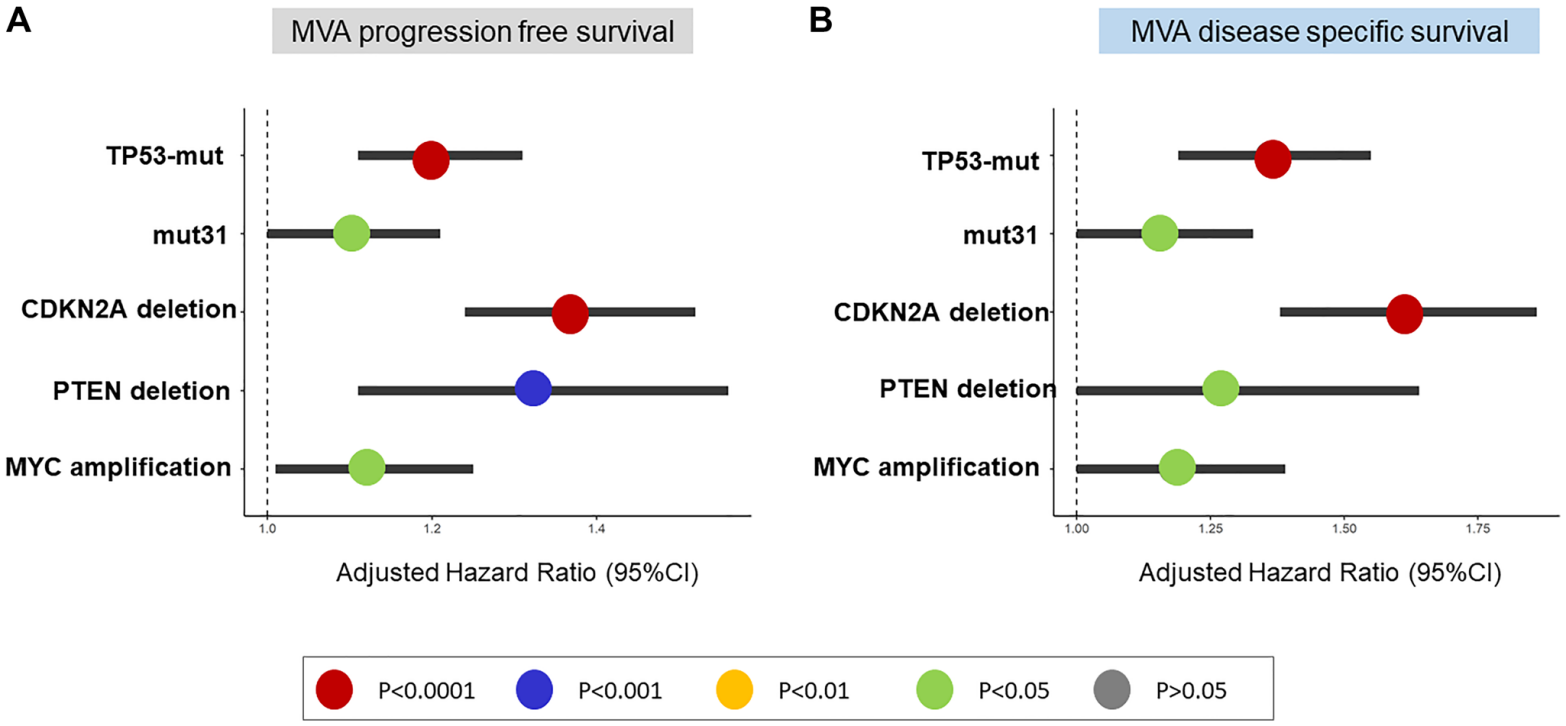
Multivariable analysis (MVA) of TP53-mutations, mut31, CDKN2A-deletion, PTEN-deletion, and MYC-amplification in Pan-Cancer TCGA. (**A**, **B**). The five genomic events are independent predictors of PFS and DSS.

In MVA model adjusting for TP53 mutations, mut31, CDKN2A deletion, PTEN deletion and MYC amplification, all five alterations were independently prognostic of PFS and DSS (*P* < 0.05 for all, Figure 4A, 4B).

### Prognostic impact of common genomic alterations in tumor type (Pan-Cancer TCGA cohort)

On UVA, TP53 was prognostic for PFS in seven cancers (ACC, HNSC, LUSC, PAAD, PRAD, THYM, UCEC) and for DSS in nine cancers (ACC, KICH, KIRC, LIHC, LUAD, LUSC, PAAD, THYM, UCEC) (Supplementary Table 6; worse prognosis for all cancer types except LUSC). Mut31 was associated with worse PFS in ACC and worse DSS in ACC, ESCA, CESC, and KICH (Supplementary Table 7). On MVA, TP53 was prognostic for PFS in five cancers (HNSC, PAAD, PRAD, THYM, UCEC) and DSS in seven cancers (ACC, KIRC, LIHC, LUAD, LUSC, PAAD, UCEC) (Supplementary Table 8).

### Independent validation of the prognostic impact of common genomic alterations in cancer (MSK-IMPACT cohort)

In the MSK-IMPACT (validation) cohort, TP53 mutations occurred at a frequency of 47% in patients with non-metastatic disease and 48% in patients with metastatic disease. In the MSK-IMPACT cohort, 74% had at least one mut31 mutation in metastatic and 71% in non-metastatic disease, and 78% of patients with TP53 mutations had an additional mutation in at least one of the mut31 genes in metastatic and non-metastatic disease. Furthermore, CDKN2A deletion, PTEN deletion, and MYC amplification occurred more frequently in metastatic (7.4%, 3.3%, and 6.7%, respectively) than non-metastatic disease (4.2%, 1.6%, and 3.8%, respectively).

**Figure 5:**
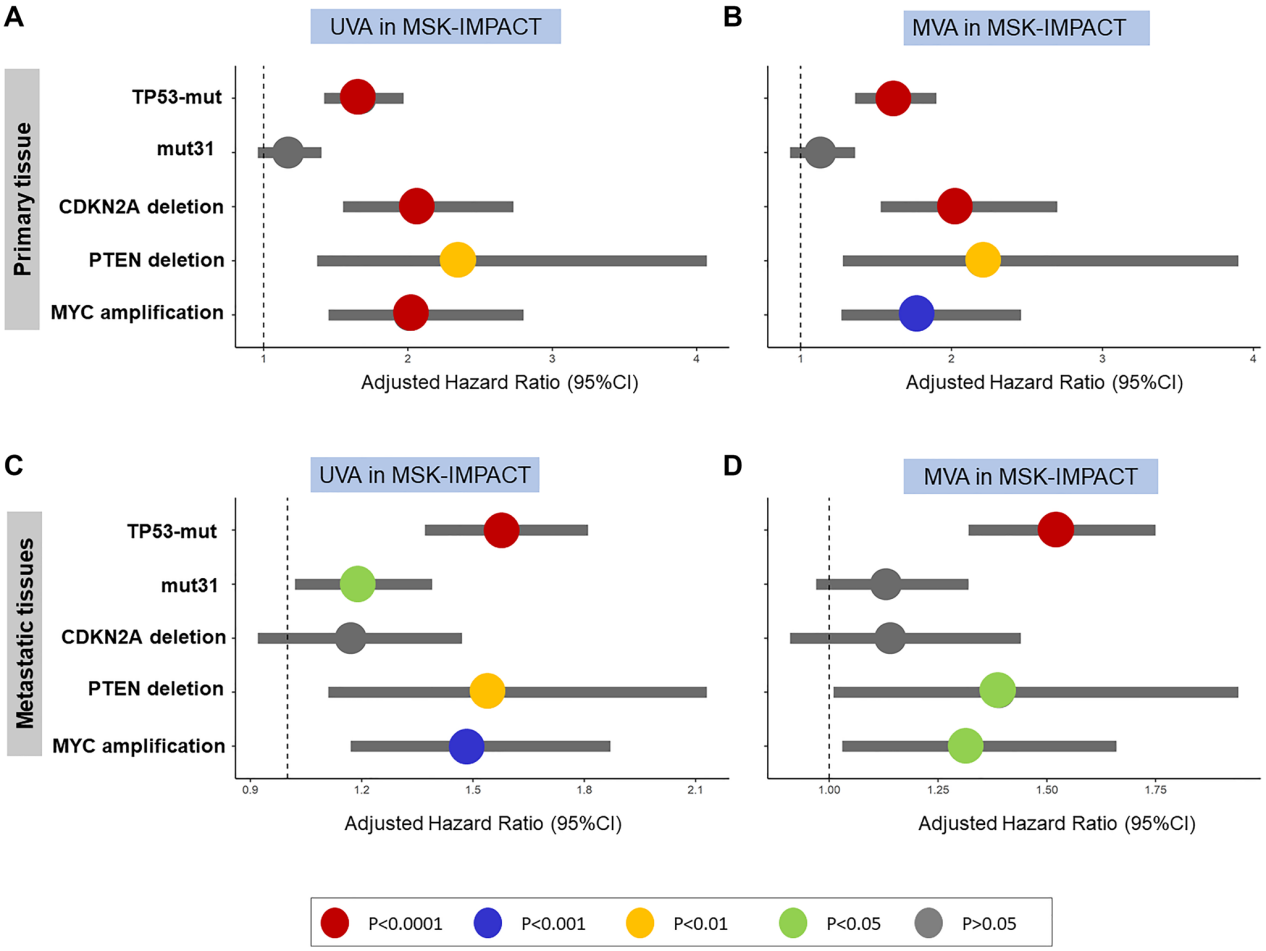
In MSK-IMPACT validation cohort, TP53-mutations, CDKN2A-deletion, PTEN-deletion, and MYC-amplifications are independent predictors of poor OS in primary tumors in (**A**) UVA and (**B**) MVA. Only TP53- mutations, PTEN-deletion, and MYC-amplification are prognostic of OS in metastatic tumors in (**C**) UVA and (**D**) MVA.

On both UVA and MVA, TP53 mutations, CDKN2A deletion, PTEN deletion, and MYC amplification were prognostic for poor OS in patients with non-metastatic disease, but mut31 was not (Figure 5A, 5B). In the metastatic disease setting, TP53 mutations, mut31, PTEN deletion, and MYC amplification were prognostic of poor OS on UVA, while TP53 mutations, PTEN deletion, and MYC amplification were prognostic of poor OS on MVA (Figure 5C, 5D).

### Comparison of results between the TCGA and MSK-IMPACT cohorts

TP53 was a significant biomarker in both cohorts, as it was shown to be independently associated with worse outcomes for both PFS and DSS in TCGA (*p*<), and significantly associated with overall survival in MSK-IMPACT (*p* < 0.0001). These results were consistent across both UVA and MVA analysis.

Mut31, on the other hand, was not as significant of a biomarker as TP53. It was prognostic for DSS and PFS by itself (*p* < 0,0001), but due to the large overlap with TP53 (81% of TP53 mutated samples also had a mut31 mutation), it was not a predictor for DSS or PFS in the TCGA cohort. Additionally, in the MSK-IMPACT cohort, mut31 was only significantly associated with poor OS on UVA in metastatic disease.

CN alterations showed consistent data for the 3 main markers: CDKN2A deletion, PTEN deletion and MYC amplification. These were prognostic of worse PFS and DSS in the TCGA cohorts both on UVA and MVA, which stayed consistent when adjusting for the mutational variables (TP53 and mut31) (*p* < 0,05 and below). In MSK-IMPACT, these 3 CN alterations were all significantly associated with worse OS in non-metastatic patients, with all but CDKN2A deletion significant in metastatic patients (*p* < 0.05 and below).

## DISCUSSION

Application of clinical genomics to diagnosis, prognosis, and treatment has become routine in many cancers and has been adopted by several cancer institutions in clinical practice. MSKCC, for example, developed and implemented MSK-IMPACT, an NGS panel capable of detecting all protein-coding mutations. MSK-IMPACT has prospectively sequenced tumors from more than 10,000 cancer patients, spanning a vast array of solid tumor types; these data are publicly available on http://www.cbioportal.org. In the current study, we utilized data from both the MSK-IMPACT cohort and the Pan-Cancer TCGA cohort of more than 8,000 patients with PFS and DSS outcomes to conduct the first and largest comprehensive analysis of prognostic genomic alterations across cancers. The data presented here offer several novel insights. First, TP53 is the most prognostic mutation regardless of the mutational status or copy number alterations of the other commonly altered genes, while other mutations were not prognostic of poor outcomes. Having any mutation in mut31 is slightly associated with poor clinical outcomes after adjusting for TP53, suggesting that TP53 mutations have the most impactful prognostic implications, and that gaining additional mutation is not increasing the risk of poor clinical outcome in patients with TP53 mutations. This result was validated in two large pan-cancer cohorts with different clinical outcomes (PFS, DSS, OS).

Second, CDKN2A deletion, PTEN deletion, and MYC amplification are independent predictors of poor outcome when adjusting for TP53, suggesting that these alterations alone are sufficient drivers of aggressive cancers. CDKN2A deletion is associated with the highest risk of poor PFS and DSS in MVA models including the four events. How CDKN2A deletion drives aggressive cancer and how it might cross-talk with TP53 warrant investigation. Finally, we used OS data from the MSK-IMPACT to demonstrate that the four alterations remained independent predictors of poor OS in primary and metastatic cancer. This finding indicates that primary tumors with any of these alterations or any combinations of them should have more aggressive treatment. Clinical trials enriching for these alterations could identify treatment options for patients harbouring one or more of these alterations.

There were several limitations in this study. One limitation of this study is that non-silent mutations were treated equally without being further sub-grouped based on their function or location. Adjustment for molecular heterogeneity was very difficult given the available variables in these datasets. Additionally, the lack of clinical variables such as tumor stage and size in some cohorts, especially in MSK-IMPACT, precluded analysis which could identify interesting results with disaggregation among cancer types. The two cohorts used different endpoints (PFS and DSS vs. OS) which allowed for the independent validation of prognostic value, but having the same endpoints would be a stronger form of validation. It is also important to note that some mut31 genes have a larger influence on outcomes in certain cancers over others. While some of these genes are specific for certain individual cancers, a limitation of this study is that we are pooling all cancers together, thus not adjusting for molecular heterogeneity. Future work should continue to investigate the prognostic values of mutations across cancers.

## MATERIALS AND METHODS

### Patient cohorts

The Pan-Cancer TCGA cohort was used to query genomic alteration data in 9,104 patients across 28 cancer types; 7,465 of these patients had clinical outcome data for survival analyses described below. The MSK-IMPACT pan-cancer cohort (*n* = 7,051) was used for independent validation of survival analyses in primary (*n* = 3,684) and metastatic cancer (*n* = 3,367). Clinical outcome data were downloaded from the TCGA Pan-Cancer Clinical Data Resource [11]. MSK-IMPACT data (mutations, copy number, and survival data) were downloaded from the cBioPortal for Cancer Genomics (http://www.cbioportal.org/).

### Common pan-cancer genomic alterations

Non-silent somatic mutation data and copy number (CN) alterations for the TCGA pan-cancer cohort were downloaded from the UCSC Xenabrowser (https://xenabrowser.net). CN were defined based on GISTC2.0 scores; samples with scores less than −0.8 were defined as CN loss, and tumors with scores greater than 1 were defined as CN amplification. There were 32 mutated genes and 15 CN-altered genes with alteration frequency greater than or equal to 4% (Supplementary Table 1) were used for survival analyses. Because the other mutations in the 32 gene set were much less frequent than TP53, they were pooled into one group (mut31). Mut31 was annotated as 1 if there was a mutation in any of the 31 genes and 0 if there was no mutation in any of the genes.

### Prognostic impact of common genomic alterations in cancer

The primary study endpoints were progression free survival (PFS) and disease specific survival (DSS) in TCGA and overall survival (OS) for MSK-IMPACT. The primary independent variables of interest were genomic alterations including TP53 mutations, a mutation in any other commonly mutated gene (mut31), and CN alterations. Cox regression analyses defined adjusted hazard ratios (AHRs) for the aforementioned endpoints in the TCGA cohort. Univariable analyses were conducted on the aforementioned genomic alterations of interest. To determine whether mut31 or CN alterations were prognostic independent of TP53 mutation, multivariable analyses were repeated with TP53 mutation status, mut31 status and CN status in one model with cancer type as stratified covariable. The aforementioned Cox regression analyses were repeated in the MSK-IMPACT cohort in each disease state (metastatic versus non-metastatic) separately. Lastly, all survival analyses were stratified by tumor type in the Cox regression models.

### Statistical analysis

Cox proportional hazard analyses defined AHRs and 95% confidence intervals (CIs), as described above. Statistical testing was two-sided with *P* < 0.05 considered significant.

## SUPPLEMENTARY MATERIALS


