# Supplementary material for: Genomic alterations predictive of poor clinical outcomes in pan-cancer
Source: Oncotarget. 2022 Sep 28;13:1069–77. doi: 10.18632/oncotarget.28276 (PMC9518688; doi:10.18632/oncotarget.28276)
Supplement: Supplementary file 1 [file oncotarget-13-28276-s001.pdf]

# Genomic alterations predictive of poor clinical outcomes in pan-cancer

## SUPPLEMENTARY MATERIALS

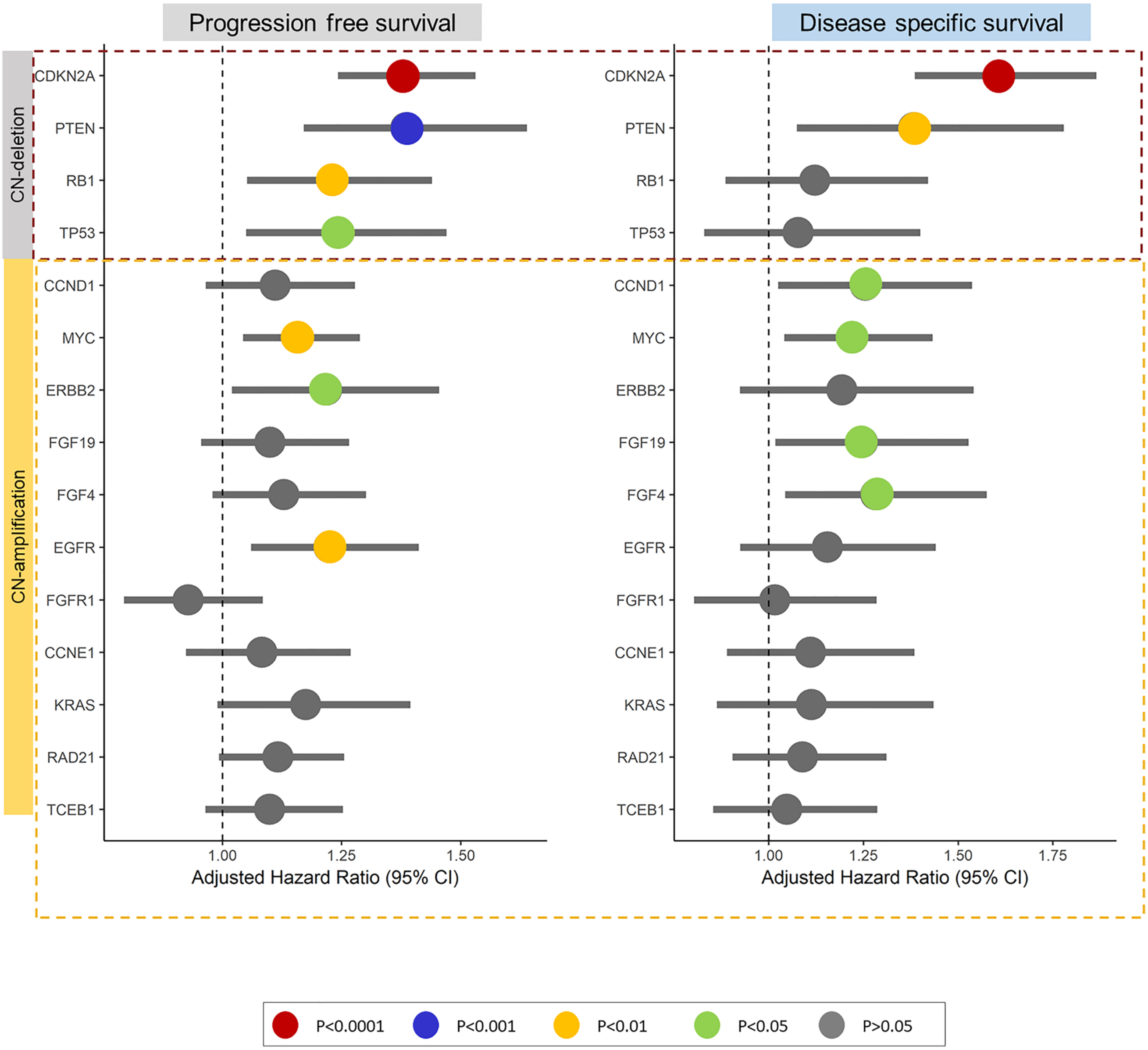


**Supplementary Figure 1: Adjusted hazard ratios from stratified Cox regression model predict PFS and DSS in the Pan-Cancer TCGA cohort for the copy number alterations.**

**Supplementary Table 1: Frequency of most altered genes in TCGA**

| Gene | Mutation frequency (%) | Gene | Amplification frequency | Gene | Deletion frequency |
|---|---|---|---|---|---|
| TP53 | 35.9 | CCND1 | 7.206965841 | CDKN2A | 10.9 |
| PIK3CA | 13.1 | MYC | 13.07434695 | PTEN | 4.5 |
| KMT2D | 9.6 | ERBB2 | 4.259879437 | RB1 | 5.3 |
| KMT2C | 8.8 | FGF19 | 7.126590757 | TP53 | 4.5 |
| PTEN | 8.2 | FGF4 | 5.653047555 | | |
| BRAF | 8.1 | EGFR | 7.032819826 | | |
| ARID1A | 7.9 | FGFR1 | 6.068318821 | | |
| KRAS | 7.4 | CCNE1 | 4.782317482 | | |
| APC | 7 | KRAS | 5.157401206 | | |
| FAT1 | 6.8 | RAD21 | 10.34159411 | | |
| ATRX | 6.3 | TCEB1 | 8.211654387 | | |
| NF1 | 5.8 | | | | |
| IDH1 | 5.7 | | | | |
| ATM | 5.2 | | | | |
| PTPRD | 5 | | | | |
| PTPRT | 5 | | | | |
| ZFHX3 | 4.9 | | | | |
| GRIN2A | 4.7 | | | | |
| ERBB4 | 4.5 | | | | |
| FBXW7 | 4.4 | | | | |
| KMT2A | 4.4 | | | | |
| CREBBP | 4.4 | | | | |
| RB1 | 4.3 | | | | |
| ROS1 | 4.3 | | | | |
| CDKN2A | 4.3 | | | | |
| NOTCH1 | 4.3 | | | | |
| BRCA2 | 4.2 | | | | |
| SETD2 | 4.2 | | | | |
| PBRM1 | 4.2 | | | | |
| CTNNB1 | 4.1 | | | | |
| EP300 | 4.1 | | | | |
| SPEN | 4 | | | | |

**Supplementary Table 2: Hazard ratios of mutations across cancers for PFS and DSS end points**

| PFS | | | | DSS | | | |
|---|---|---|---|---|---|---|---|
| **Gene** | **HR** | **95 % CI** | ***p* value** | **Gene** | **HR** | **95 % CI** | ***p* value** |
| TP53 | 1.225031 | 1.122–1.337 | 5.92E-06 | TP53 | 1.382484 | 1.212–1.577 | 1.41E-06 |
| IDH1 | 1.304839 | 0.962–1.771 | 0.0875 | IDH1 | 1.245806 | 0.781–1.987 | 0.356066 |
| KRAS | 1.109431 | 0.96–1.282 | 0.158273 | KRAS | 1.132095 | 0.912–1.405 | 0.260345 |
| ZFHX3 | 1.063985 | 0.895–1.265 | 0.482219 | ZFHX3 | 1.120246 | 0.861–1.457 | 0.397457 |
| PTPRT | 1.054687 | 0.9–1.236 | 0.51099 | CDKN2A | 1.100855 | 0.857–1.413 | 0.450969 |
| APC | 1.026764 | 0.864–1.221 | 0.764789 | NF1 | 1.066066 | 0.838–1.355 | 0.601568 |
| KMT2D | 0.995775 | 0.883–1.123 | 0.944957 | KMT2C | 1.062313 | 0.878–1.285 | 0.533102 |
| SETD2 | 0.995654 | 0.823–1.205 | 0.964263 | GRIN2A | 1.058644 | 0.804–1.393 | 0.684133 |
| PTPRD | 0.993604 | 0.84–1.176 | 0.94047 | FBXW7 | 1.034509 | 0.805–1.33 | 0.791042 |
| PBRM1 | 0.988199 | 0.82–1.191 | 0.900967 | KMT2D | 1.033606 | 0.867–1.232 | 0.711711 |
| KMT2C | 0.984502 | 0.866–1.119 | 0.811587 | PIK3CA | 1.027947 | 0.866–1.22 | 0.752635 |
| BRAF | 0.976171 | 0.799–1.192 | 0.813291 | APC | 1.025591 | 0.78–1.348 | 0.856104 |
| CDKN2A | 0.972453 | 0.821–1.151 | 0.74577 | KMT2A | 0.981995 | 0.754–1.279 | 0.892904 |
| KMT2A | 0.968384 | 0.811–1.156 | 0.721706 | PTPRT | 0.981538 | 0.774–1.246 | 0.878139 |
| FBXW7 | 0.963884 | 0.808–1.15 | 0.682993 | FAT1 | 0.968347 | 0.787–1.192 | 0.761815 |
| NF1 | 0.963586 | 0.815–1.139 | 0.66372 | EP300 | 0.96263 | 0.744–1.246 | 0.77233 |
| FAT1 | 0.95319 | 0.83–1.095 | 0.49895 | PBRM1 | 0.949485 | 0.716–1.258 | 0.718386 |
| ATM | 0.948548 | 0.8–1.124 | 0.542017 | PTPRD | 0.915331 | 0.705–1.188 | 0.505794 |
| GRIN2A | 0.907186 | 0.749–1.098 | 0.317994 | BRCA2 | 0.911237 | 0.701–1.185 | 0.488437 |
| PIK3CA | 0.897304 | 0.796–1.011 | 0.075221 | ATM | 0.903731 | 0.688–1.188 | 0.467821 |
| ARID1A | 0.893582 | 0.777–1.027 | 0.113839 | CTNNB1 | 0.896134 | 0.674–1.192 | 0.451308 |
| RB1 | 0.879344 | 0.713–1.085 | 0.22982 | CREBBP | 0.889547 | 0.666–1.189 | 0.428993 |
| CTNNB1 | 0.864672 | 0.715–1.046 | 0.133503 | NOTCH1 | 0.888829 | 0.677–1.167 | 0.396743 |
| EP300 | 0.862625 | 0.716–1.039 | 0.118744 | RB1 | 0.864808 | 0.618–1.21 | 0.396209 |
| ERBB4 | 0.84905 | 0.705–1.022 | 0.083759 | BRAF | 0.86369 | 0.61–1.223 | 0.408928 |
| CREBBP | 0.845972 | 0.696–1.029 | 0.093472 | ERBB4 | 0.829615 | 0.62–1.109 | 0.207677 |
| PTEN | 0.845842 | 0.705–1.014 | 0.070747 | SETD2 | 0.810859 | 0.585–1.123 | 0.207579 |
| ROS1 | 0.844173 | 0.694–1.027 | 0.091053 | ARID1A | 0.803925 | 0.649–0.996 | 0.045379 |
| BRCA2 | 0.815175 | 0.675–0.984 | 0.033335 | ROS1 | 0.796141 | 0.595–1.064 | 0.123988 |
| SPEN | 0.794841 | 0.65–0.972 | 0.02547 | PTEN | 0.763367 | 0.572–1.018 | 0.065932 |
| NOTCH1 | 0.78143 | 0.649–0.941 | 0.009322 | ATRX | 0.701948 | 0.548–0.898 | 0.004934 |
| ATRX | 0.761928 | 0.652–0.89 | 0.000616 | SPEN | 0.679095 | 0.49–0.941 | 0.020057 |

HR were from COX hazard models stratfied by cancer types

**Supplementary Table 3: Hazard ratios of mutations across cancers when adjusting for TP53 in a cancer stratified COX regression**

| PFS | | | | DSS | | | |
|---|---|---|---|---|---|---|---|
| **Gene** | **HR** | **95% CI** | ***p* value** | **Gene** | **HR** | **95% CI** | ***p* value** |
| KRAS | 1.11919 | 0.968–1.294 | 0.128323 | KRAS | 1.139264 | 0.916–1.417 | 0.242084 |
| PIK3CA | 0.913934 | 0.811–1.03 | 0.140219 | PIK3CA | 1.065293 | 0.897–1.265 | 0.470836 |
| APC | 1.015703 | 0.854–1.208 | 0.860316 | APC | 1.015737 | 0.772–1.336 | 0.911106 |
| ARID1A | 0.917929 | 0.798–1.055 | 0.228895 | ARID1A | 0.834719 | 0.674–1.033 | 0.096875 |
| KMT2D | 0.99449 | 0.882–1.121 | 0.928181 | KMT2D | 1.032522 | 0.867–1.23 | 0.720308 |
| PTEN | 0.873511 | 0.729–1.046 | 0.141733 | PTEN | 0.797602 | 0.601–1.059 | 0.118244 |
| KMT2C | 0.978578 | 0.861–1.113 | 0.741021 | KMT2C | 1.040549 | 0.86–1.259 | 0.682134 |
| NF1 | 0.957855 | 0.81–1.132 | 0.613892 | NF1 | 1.051856 | 0.827–1.338 | 0.680291 |
| BRAF | 0.986602 | 0.808–1.205 | 0.895004 | BRAF | 0.874097 | 0.617–1.238 | 0.448898 |
| FAT1 | 0.944168 | 0.822–1.085 | 0.418094 | FAT1 | 0.953792 | 0.775–1.174 | 0.655964 |
| RB1 | 0.863008 | 0.7–1.065 | 0.168957 | RB1 | 0.837229 | 0.599–1.171 | 0.299245 |
| ZFHX3 | 1.063604 | 0.895–1.264 | 0.484136 | ZFHX3 | 1.121548 | 0.863–1.458 | 0.391861 |
| ATRX | 0.735268 | 0.629–0.86 | 0.000116 | ATRX | 0.655513 | 0.511–0.84 | 0.000858 |
| ATM | 0.969331 | 0.818–1.149 | 0.719463 | ATM | 0.926404 | 0.705–1.218 | 0.583467 |
| PTPRT | 1.049718 | 0.896–1.23 | 0.549061 | PTPRT | 0.98324 | 0.775–1.247 | 0.88908 |
| CDKN2A | 0.933219 | 0.788–1.106 | 0.42455 | CDKN2A | 1.041373 | 0.811–1.338 | 0.75112 |
| SETD2 | 1.020265 | 0.843–1.235 | 0.836674 | SETD2 | 0.832236 | 0.601–1.153 | 0.269469 |
| CREBBP | 0.84334 | 0.694–1.025 | 0.087318 | CREBBP | 0.881986 | 0.66–1.178 | 0.395692 |
| PTPRD | 0.977647 | 0.826–1.157 | 0.792594 | PTPRD | 0.890818 | 0.686–1.156 | 0.384964 |
| NOTCH1 | 0.780857 | 0.648–0.941 | 0.009155 | NOTCH1 | 0.899688 | 0.685–1.182 | 0.447427 |
| BRCA2 | 0.814772 | 0.675–0.983 | 0.03285 | BRCA2 | 0.908182 | 0.698–1.181 | 0.472869 |
| PBRM1 | 0.992174 | 0.823–1.196 | 0.934343 | PBRM1 | 0.949688 | 0.717–1.259 | 0.719464 |
| KMT2A | 0.966564 | 0.81–1.154 | 0.706212 | KMT2A | 0.976183 | 0.749–1.272 | 0.858375 |
| GRIN2A | 0.903513 | 0.746–1.094 | 0.298257 | GRIN2A | 1.059182 | 0.805–1.394 | 0.681567 |
| ROS1 | 0.831088 | 0.683–1.012 | 0.064949 | ROS1 | 0.77322 | 0.578–1.034 | 0.082682 |
| SPEN | 0.792512 | 0.648–0.969 | 0.023622 | SPEN | 0.676132 | 0.488–0.937 | 0.018694 |
| CTNNB1 | 0.892186 | 0.738–1.079 | 0.240132 | CTNNB1 | 0.945048 | 0.71–1.258 | 0.698428 |
| ERBB4 | 0.838741 | 0.697–1.01 | 0.063083 | ERBB4 | 0.816306 | 0.61–1.091 | 0.170902 |
| EP300 | 0.865729 | 0.719–1.042 | 0.128069 | EP300 | 0.962933 | 0.744–1.247 | 0.774418 |
| FBXW7 | 0.962223 | 0.806–1.148 | 0.669049 | FBXW7 | 1.031087 | 0.802–1.326 | 0.811331 |
| IDH1 | 1.295045 | 0.954–1.757 | 0.096998 | IDH1 | 1.219048 | 0.765–1.942 | 0.404635 |

**Supplementary Table 4: Adjusted Hazard ratios of gaining additional mutations in patients with TP53 mutations (TP53-1)**

| | PFS | | DSS | |
|---|---|---|---|---|
| | **HR** | ***p*** | **HR** | ***p*** |
| mut31-0 (*n* = 511) | ref | | ref | |
| mut31-1 (*n* = 832) | 1.03 [0.87–1.22] | 0.68 | 1.2 [0.93–1.53] | 0.14 |
| mut31-2 (*n* = 611) | 0.89 [0.74–1.08] | 0.24 | 1.12 [0.85–1.46] | 0.39 |
| mut31-3 to 5 (*n* = 513) | 1.02 [0.83–1.25] | 0.82 | 1.41 [1.06–1.88] | 0.01 |
| mut31-5 and more (*n* = 259) | 0.89 [0.7–1.13] | 0.35 | 0.86 [0.6–1.24] | 0.43 |

**Supplementary Table 5: Hazard ratios ofcopy number alterations across cancers when adjusting for TP53 mutations in a cancer stratified COX regression**

| | PFS | | | | DSS | | |
|---|---|---|---|---|---|---|---|
| | **HR** | **95% CI** | ***p* value** | | **HR** | **95% CI** | ***p* value** |
| CDKN2A-del | 1.383329495 | 1.246–1.536 | 1.11E-09 | CDKN2A-del | 1.621901 | 1.399–1.881 | 1.54E-10 |
| PTEN-del | 1.339068119 | 1.131–1.585 | 0.000703832 | PTEN-del | 1.32721 | 1.031–1.709 | 0.028066 |
| RB1-del | 1.195442267 | 1.021–1.4 | 0.026526427 | RB1-del | 1.082407 | 0.854–1.372 | 0.513189 |
| TP53-del | 1.216452174 | 1.028–1.44 | 0.022893022 | TP53-del | 1.049383 | 0.808–1.364 | 0.718377 |
| CCND1-AMPL | 1.090740461 | 0.948–1.256 | 0.226309912 | CCND1-AMPL | 1.218658 | 0.995–1.493 | 0.056546 |
| MYC-AMPL | 1.126732769 | 1.013–1.253 | 0.027817958 | MYC-AMPL | 1.173577 | 1.001–1.378 | 0.049834 |
| ERBB2-AMPL | 1.187453568 | 0.994–1.418 | 0.058173078 | ERBB2-AMPL | 1.155825 | 0.895–1.493 | 0.267144 |
| FGF19-AMPL | 1.079792238 | 0.938–1.244 | 0.286630636 | FGF19-AMPL | 1.209818 | 0.987–1.484 | 0.06723 |
| FGF4-AMPL | 1.108455279 | 0.961–1.278 | 0.157251135 | FGF4-AMPL | 1.241639 | 1.01–1.527 | 0.040168 |
| EGFR-AMPL | 1.184066293 | 1.025–1.367 | 0.021315389 | EGFR-AMPL | 1.099205 | 0.88–1.373 | 0.404659 |
| FGFR1-AMPL | 0.908547472 | 0.777–1.062 | 0.229185668 | FGFR1-AMPL | 0.983884 | 0.778–1.245 | 0.892225 |
| CCNE1-AMPL | 1.047613944 | 0.893–1.228 | 0.566872084 | CCNE1-AMPL | 1.063017 | 0.852–1.326 | 0.588526 |
| KRAS-AMPL | 1.161216457 | 0.979–1.378 | 0.087014954 | KRAS-AMPL | 1.095329 | 0.85–1.412 | 0.482508 |
| RAD21-AMPL | 1.088878725 | 0.968–1.225 | 0.157278927 | RAD21-AMPL | 1.054041 | 0.875–1.269 | 0.578799 |
| TCEB1-AMPL | 1.079455521 | 0.947–1.231 | 0.252532737 | TCEB1-AMPL | 1.02335 | 0.833–1.257 | 0.825703 |

**Supplementary Table 6: Hazard ratios of TP53 mutations across individual cancers for PFS and DSS**

| TP53/PFS | | | | TP53/DSS | | | |
|---|---|---|---|---|---|---|---|
| **Cancer** | **HR** | **95% CI** | ***p* value** | **Cancer** | **HR** | **95% CI** | ***p* value** |
| ACC | 2.587748713 | 1.305–5.133 | 0.006509838 | ACC | 4.09544963 | 1.76–9.528 | 0.001065495 |
| BLCA | 1.044524161 | 0.805–1.355 | 0.742963286 | BLCA | 0.991378461 | 0.692–1.42 | 0.962292492 |
| BRCA | 1.102109557 | 0.785–1.548 | 0.574893719 | BRCA | 1.38220396 | 0.788–2.425 | 0.259251348 |
| CHOL | 0.722718347 | 0.169–3.099 | 0.661939792 | CHOL | 0.798540364 | 0.172–3.698 | 0.773603257 |
| ESCA | 1.290250684 | 0.746–2.231 | 0.361881732 | ESCA | 2.213114644 | 0.795–6.161 | 0.128314897 |
| DLBC | 0.509191686 | 0.064–4.055 | 0.523762411 | DLBC | 4.28E-09 | NA | 0.99939273 |
| CESC | 1.300117836 | 0.598–2.826 | 0.507557228 | CESC | 1.801943135 | 0.766–4.237 | 0.176994701 |
| COAD | 1.31075744 | 0.868–1.979 | 0.198046033 | COAD | 1.520492664 | 0.723–3.197 | 0.269153191 |
| KICH | 2.481161226 | 0.799–7.705 | 0.115991209 | KICH | 5.711940038 | 1.106–29.49 | 0.037468953 |
| ESCA | 1.290250684 | 0.746–2.231 | 0.361881732 | ESCA | 2.213114644 | 0.795–6.161 | 0.128314897 |
| HNSC | 1.564622398 | 1.171–2.091 | 0.002498462 | HNSC | 1.418746118 | 0.947–2.126 | 0.090060634 |
| KIRC | 2.044194392 | 0.9–4.645 | 0.087785105 | KIRC | 3.543275977 | 1.283–9.787 | 0.014673104 |
| MESO | 1.052923178 | 0.563–1.97 | 0.87182076 | MESO | 0.984745603 | 0.468–2.071 | 0.967663654 |
| KIRP | 1.648165557 | 0.402–6.762 | 0.487852203 | KIRP | 3.520347505 | 0.831–14.909 | 0.087450654 |
| LIHC | 1.253604867 | 0.94–1.671 | 0.123404569 | LIHC | 1.842695495 | 1.139–2.982 | 0.012790677 |
| LUAD | 1.090914097 | 0.851–1.398 | 0.491595119 | LUAD | 1.488560231 | 1.02–2.172 | 0.039010192 |
| LUSC | 0.662320809 | 0.477–0.92 | 0.014141677 | LUSC | 0.435792712 | 0.26–0.731 | 0.001627828 |
| PAAD | 1.723757533 | 1.158–2.567 | 0.007348955 | PAAD | 1.654403598 | 1.004–2.727 | 0.048379365 |
| READ | 0.985969329 | 0.338–2.878 | 0.979373653 | READ | 80641519.93 | NA | 0.998687859 |
| PRAD | 1.918226649 | 1.169–3.147 | 0.009900327 | PRAD | 3.840778829 | 0.612–24.096 | 0.150936168 |
| SKCM | 0.732764045 | 0.226–2.374 | 0.604127408 | SKCM | 1.00841164 | 0.23–4.414 | 0.99112721 |
| TGCT | 3.02E-07 | NA | 0.997074756 | TGCT | 8.21E-07 | NA | 0.999090235 |
| SARC | 1.226659604 | 0.867–1.736 | 0.24895694 | SARC | 1.164594029 | 0.725–1.872 | 0.528987191 |
| STAD | 1.017138243 | 0.757–1.367 | 0.910294473 | STAD | 1.03987443 | 0.69–1.567 | 0.851713991 |
| THYM | 11.55004737 | 3.654–36.51 | 3.09E-05 | THYM | 16.34936387 | 1.358–196.804 | 0.027725277 |
| THCA | 3.00E-07 | NA | 0.995087571 | THCA | 3.01E-07 | NA | 0.998537819 |
| UCEC | 1.832945912 | 1.274–2.638 | 0.001102487 | UCEC | 3.797237174 | 2.037–7.079 | 2.69E-05 |
| UCS | 0.556180058 | 0.216–1.429 | 0.223070517 | UCS | 0.402976867 | 0.139–1.164 | 0.093186415 |
| UVM | NA | NA | NA | UVM | NA | NA | NA |

**Supplementary Table 7: Hazard ratios of mut31 mutations across individual cancers for PFS and DSS**

| mut31/PFS | | | | mut31/DSS | | | |
|---|---|---|---|---|---|---|---|
| Cancer | HR | 95% CI | *p* value | Cancer | HR | 95% CI | *p* value |
| ACC | 2.224899027 | 1.201–4.122 | 0.011016136 | ACC | 3.339319622 | 1.539–7.244 | 0.002274029 |
| BLCA | 0.953136172 | 0.691–1.315 | 0.769967013 | BLCA | 1.139125582 | 0.698–1.86 | 0.602606213 |
| BRCA | 0.880235279 | 0.616–1.258 | 0.483940294 | BRCA | 0.978738229 | 0.518–1.849 | 0.94722367 |
| CHOL | 1.640558334 | 0.641–4.2 | 0.302063801 | CHOL | 2.187612601 | 0.687–6.967 | 0.185318016 |
| ESCA | 1.301708601 | 0.888–1.907 | 0.176022123 | ESCA | 2.052273854 | 1.103–3.82 | 0.023321256 |
| DLBC | 1.082931375 | 0.309–3.793 | 0.90086156 | DLBC | 311344511.8 | NA | 0.999204142 |
| CESC | 1.626588032 | 0.941–2.812 | 0.081581387 | CESC | 2.957387447 | 1.258–6.95 | 0.0128701 |
| COAD | 0.386153187 | 0.053–2.798 | 0.346345117 | COAD | 3317637.002 | NA | 0.997540784 |
| KICH | 3.566711033 | 1.12–11.355 | 0.031376623 | KICH | 5.651431798 | 1.25–25.555 | 0.024473393 |
| HNSC | 1.056175584 | 0.788–1.415 | 0.714276668 | HNSC | 1.555946157 | 0.982–2.466 | 0.059944442 |
| KIRC | 1.231599146 | 0.845–1.795 | 0.278766059 | KIRC | 1.03139321 | 0.605–1.758 | 0.909559139 |
| MESO | 1.056589747 | 0.643–1.737 | 0.82810239 | MESO | 0.74900408 | 0.404–1.388 | 0.358691127 |
| KIRP | 1.347961551 | 0.818–2.222 | 0.241538737 | KIRP | 0.886510886 | 0.41–1.918 | 0.759667461 |
| LIHC | 0.892659213 | 0.679–1.173 | 0.415318586 | LIHC | 1.120963765 | 0.699–1.797 | 0.635391879 |
| LUAD | 1.221580271 | 0.788–1.894 | 0.371152444 | LUAD | 1.172668299 | 0.611–2.249 | 0.631718446 |
| LUSC | 0.947114638 | 0.39–2.298 | 0.904374655 | LUSC | 0.933868275 | 0.229–3.803 | 0.923917328 |
| PAAD | 0.876211181 | 0.543–1.414 | 0.588610322 | PAAD | 1.045000152 | 0.558–1.959 | 0.890777078 |
| PRAD | 1.493022437 | 0.978–2.279 | 0.063202373 | PRAD | 1.293668677 | 0.205–8.18 | 0.784353504 |
| SKCM | 0.606537206 | 0.304–1.209 | 0.155275944 | SKCM | 0.669383164 | 0.253–1.771 | 0.418713838 |
| TGCT | 1.212166928 | 0.563–2.611 | 0.623054277 | TGCT | 0.287992374 | 0.026–3.195 | 0.310633574 |
| SARC | 1.392333079 | 0.974–1.991 | 0.069538933 | SARC | 1.197465275 | 0.728–1.969 | 0.477540587 |
| STAD | 0.815496826 | 0.6–1.109 | 0.19357762 | STAD | 0.647593369 | 0.428–0.979 | 0.039505735 |
| THYM | 2.036872973 | 0.842–4.93 | 0.114669855 | THYM | 5.25361152 | 0.738–37.392 | 0.097570523 |
| THCA | 1.250277916 | 0.716–2.183 | 0.432099314 | THCA | 0.732865247 | 0.121–4.423 | 0.734711377 |
| UCEC | 0.652416311 | 0.341–1.249 | 0.197304405 | UCEC | 0.366710382 | 0.155–0.866 | 0.022047174 |
| UCS | 1.425540475 | 0.648–3.138 | 0.378491224 | UCS | 1.389981983 | 0.56–3.45 | 0.477801633 |
| UVM | 1.045157628 | 0.36–3.035 | 0.935287619 | UVM | 0.759611936 | 0.175–3.296 | 0.713493866 |

**Supplementary Table 8: Hazard ratios of TP53 mutations in invididual cancers when adjusting for mut31 status for PFS and DSS**

| | PFS | | | | DSS | | |
|---|---|---|---|---|---|---|---|
| **Cancer** | **HR** | **95% CI** | ***p* value** | **Cancer** | **HR** | **95% CI** | ***p* value** |
| ACC | 1.964558489 | 0.909–4.248 | 0.086142074 | ACC | 2.67353354 | 1.037–6.894 | 0.041889596 |
| BLCA | 1.05465463 | 0.809–1.376 | 0.694626155 | BLCA | 0.976857017 | 0.68–1.404 | 0.899302218 |
| BRCA | 1.117599558 | 0.794–1.573 | 0.523791081 | BRCA | 1.384743205 | 0.789–2.432 | 0.257208928 |
| CHOL | 0.585340292 | 0.133–2.581 | 0.47923782 | CHOL | 0.595729156 | 0.127–2.785 | 0.510368674 |
| ESCA | 1.276637091 | 0.738–2.207 | 0.381939327 | ESCA | 2.176775921 | 0.782–6.059 | 0.136393209 |
| DLBC | 0.442799369 | 0.05–3.929 | 0.464537943 | DLBC | 7.65E-10 | NA | 0.99965565 |
| CESC | 1.150917184 | 0.525–2.524 | 0.725723995 | CESC | 1.434866848 | 0.605–3.402 | 0.412392188 |
| COAD | 1.316184046 | 0.872–1.988 | 0.191507291 | COAD | 1.518254523 | 0.722–3.192 | 0.270828253 |
| KICH | 2.287723476 | 0.733–7.142 | 0.154217772 | KICH | 4.74750021 | 0.905–24.895 | 0.065420883 |
| ESCA | 1.276637091 | 0.738–2.207 | 0.381939327 | ESCA | 2.176775921 | 0.782–6.059 | 0.136393209 |
| HNSC | 1.582645649 | 1.176–2.13 | 0.002464731 | HNSC | 1.330010594 | 0.882–2.005 | 0.173454524 |
| KIRC | 1.947961448 | 0.852–4.451 | 0.113787913 | KIRC | 3.572698704 | 1.278–9.987 | 0.01518741 |
| MESO | 1.049927525 | 0.561–1.966 | 0.878959396 | MESO | 0.964885541 | 0.458–2.031 | 0.925001983 |
| KIRP | 1.457198148 | 0.35–6.066 | 0.604851726 | KIRP | 3.928728096 | 0.879–17.569 | 0.073377879 |
| LIHC | 1.252077548 | 0.939–1.669 | 0.125526467 | LIHC | 1.84144049 | 1.138–2.979 | 0.012864779 |
| LUAD | 1.07956124 | 0.842–1.385 | 0.546802809 | LUAD | 1.483225108 | 1.016–2.165 | 0.041042602 |
| LUSC | 0.662299452 | 0.476–0.921 | 0.014286077 | LUSC | 0.433881262 | 0.258–0.729 | 0.001616688 |
| PAAD | 2.025605868 | 1.303–3.15 | 0.001725717 | PAAD | 1.834196216 | 1.043–3.227 | 0.035300134 |
| READ | 0.985969329 | 0.338–2.878 | 0.979373653 | READ | 80641519.93 | NA | 0.998687859 |
| PRAD | 1.869346762 | 1.138–3.07 | 0.013428264 | PRAD | 3.833088025 | 0.612–23.995 | 0.151050685 |
| SKCM | 0.812229398 | 0.247–2.667 | 0.731732694 | SKCM | 1.114268273 | 0.248–5.003 | 0.887710552 |
| TGCT | 3.35E-07 | NA | 0.997100274 | TGCT | 3.87E-07 | NA | 0.999136168 |
| SARC | 1.190217621 | 0.839–1.688 | 0.328723897 | SARC | 1.132890868 | 0.699–1.837 | 0.612857882 |
| STAD | 1.025743933 | 0.763–1.379 | 0.866335815 | STAD | 1.047979522 | 0.695–1.58 | 0.823008838 |
| THYM | 11.6175726 | 2.477–54.494 | 0.001870266 | THYM | 6.853670107 | 0.35–134.256 | 0.204767138 |
| THCA | 3.12E-07 | NA | 0.995099012 | THCA | 2.89E-07 | NA | 0.998533655 |
| UCEC | 1.796841866 | 1.237–2.61 | 0.002083217 | UCEC | 3.571621767 | 1.891–6.744 | 8.67E-05 |
| UCS | 0.531085288 | 0.206–1.37 | 0.19055836 | UCS | 0.425540353 | 0.145–1.252 | 0.12074364 |
| UVM | NA | NA | NA | UVM | NA | NA | NA |
